# Addressing the System-Size Dependence of the Local Approximation Error in Coupled-Cluster Calculations

**DOI:** 10.1021/acs.jpca.1c09106

**Published:** 2021-11-03

**Authors:** Ahmet Altun, Soumen Ghosh, Christoph Riplinger, Frank Neese, Giovanni Bistoni

**Affiliations:** † Max-Planck-Institut für Kohlenforschung, Kaiser-Wilhelm-Platz 1, D-45470 Mülheim an der Ruhr, Germany; ‡ FAccTs GmbH, Rolandstrasse 67, 50677 Köln, Germany

## Abstract

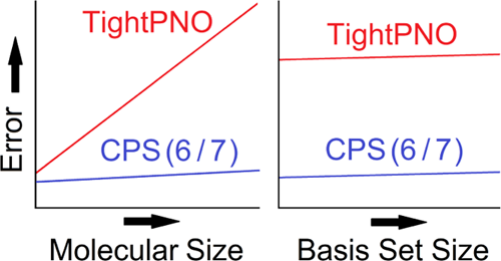

Over the last two decades, the local approximation has been successfully used to extend the range of applicability of the “gold standard” singles and doubles coupled-cluster method with perturbative triples CCSD(T) to systems with hundreds of atoms. The local approximation error grows in absolute value with the increasing system size, i.e., by increasing the number of electron pairs in the system. In this study, we demonstrate that the recently introduced two-point extrapolation scheme for approaching the complete pair natural orbital (PNOs) space limit in domain-based pair natural orbital CCSD(T) calculations drastically reduces the dependence of the error on the system size, thus opening up unprecedented opportunities for the calculation of benchmark quality relative energies for large systems.

## 1 Introduction

The coupled-cluster method with singles, doubles, and perturbative triples, i.e., CCSD(T),^1^ is generally considered as the “gold standard” of quantum chemistry.^2^ However, CCSD(T) is affordable only for very small systems since its computational cost scales as the seventh power of the system size. This stimulated the development of approximations aimed at reducing its steep scaling while retaining its great accuracy. In particular, modern local variants of CCSD(T), which exploit the rapid decay of electron correlation with the interelectronic distance, have demonstrated linear or low-order scaling.^3−5^ For example, the popular domain-based local pair natural orbital CCSD(T) method [DLPNO-CCSD(T)]^6−14^ can be used to compute energies and properties for systems as large as entire proteins.^15^

Such efficiency is achieved with the use of two main thresholds that control the size of the correlation space: *T*_CutPairs_ and *T*_CutPNO_. Pair correlation energies larger than *T*_CutPairs_ are classified as “strong pairs” and included in the coupled-cluster treatment. The remaining “weak” pairs are treated at a local MP2 level. In addition, the coupled-cluster equations are solved in an extremely compact virtual space, which is tailored for each electron pair and spanned by a small set of pair natural orbitals (PNOs).^6^ Only the PNOs with an occupation number greater than *T*_CutPNO_ are included in the correlation space of a given electron pair.

For systems with a complex electronic structure and/or for noncovalent interactions (NCIs), the conservative “TightPNO” settings^13,14^ are typically recommended for high accuracy calculations. With these settings, the *T*_CutPairs_ and *T*_CutPNO_ thresholds are set to 10^–5^ and 10^–7^, respectively. Thus, DLPNO-CCSD(T)/TightPNO typically retains about 99.9% of the canonical CCSD(T) correlation energy, as shown on many benchmark data sets.^16−21^

Since the total energy of the system is a size-extensive property, the local approximation error shows size-extensive behavior, and hence, it grows with the system size for all local methods, as demonstrated previously in many systematic studies by various groups.^22−37^ While this is not a problem in standard chemical applications, recent benchmark studies suggested that, for very large systems with the complex electronic structure, the local approximation error in DLPNO-CCSD(T) calculations (also denoted hereafter as “DLPNO” error) might become significant.^38−44^

Although the DLPNO error can be reduced in such challenging cases by tightening the *T*_CutPairs_ and/or *T*_CutPNO_ thresholds, this might lead to calculations that are computationally too demanding for large systems. This is particularly true for the *T*_CutPNO_ threshold because it determines the size of the virtual space. Hence, the computational cost of DLPNO-CCSD(T) calculations is extremely sensitive to its value. Considering that the large majority of benchmark sets used for testing the accuracy of local methods is composed of small systems, one may still question their general reliability for large systems, at least when standard thresholds are used.

Recently, we have introduced an alternative computational strategy for approaching the “complete PNO space” (CPS) limit.^45^ Correlation energies obtained with two different *T*_CutPNO_ values are extrapolated to the CPS limit using a two-point extrapolation formula
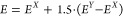
1where *E*^*X*^ and *E*^*Y*^ are the correlation energies obtained with *T*_CutPNO_ = 10^–*X*^ and 10^–*Y*^, respectively; *Y* = *X* + 1; and *E* is the correlation energy at the estimated CPS(*X*/*Y*) limit for a given basis set. For example, CPS(6/7) extrapolation yields errors in relative energies that are typically less than 0.3 kcal/mol in comparison to canonical CCSD(T) for the most challenging reactions of the GMTKN55 superset,^20^ as shown in ref (45), as well as on many other molecular systems.^46−48^ In particular, the accurate quantification of NCI energies^20,49−64^ is a very hot topic of research. On the most popular benchmark set of NCIs (S66),^65−67^ CPS(6/7) provides a mean absolute error (MAE) of only 0.03 kcal/mol at the complete basis set (CBS) limit relative to the SILVER^61^ reference values. This is below the error expected for canonical CCSD(T) for this set.^68^ Thus, CPS(6/7) has been shown to provide relative energies of benchmark quality for an extremely broad range of systems.^45^ In terms of efficiency, CPS(*X*/*Y*) extrapolation calculations were found to be faster than the corresponding *T*_CutPNO_ = 10^–(*Y*+1)^ calculations by a factor of 2.^45^

It is worth to noting here that CBS and CPS extrapolations are conceptually different approaches. CBS extrapolation corrects the energies computed with an electronic structure method for the basis set incompleteness error. Hence, the goal is to extrapolate the energy to the complete basis set limit. In contrast, CPS extrapolation corrects the error introduced in the correlation energy upon the truncation of the virtual space in local methods for a given basis set. Thus, both extrapolation schemes can and should be applied simultaneously for the calculation of benchmark quality energetics.

Notice that other extrapolation approaches to the complete virtual space have been suggested for local correlation methods.^69−71^ However, our CPS(*X*/*Y*) extrapolation scheme distinguishes itself for its simplicity and broad applicability, as it can be used without any modification to improve the accuracy of arbitrary DLPNO-CCSD(T) calculations, irrespective of the basis set size or of the PNO threshold used. It is also important to emphasize that our CPS extrapolation scheme addresses the PNO truncation error but not the domain error that arises from the truncation of the electron pair list. Hence, CPS extrapolation exploits the smooth dependence of the correlation energy with the size of the virtual space. In contrast, the previously published two-point extrapolation schemes suggested by Calbo, Aragó, and co-workers,^72^ as well as by Kállay and co-workers,^5^ try to correct several approximations used in local CCSD(T) simultaneously.

In this study, we demonstrate that CPS(6/7) extrapolation can be used to drastically reduce the dependence of the DLPNO error on the system size on a series of benchmark sets for which canonical CCSD(T) reference data are available. Hence, the DLPNO error is evaluated on (1) absolute energies of linear alkane chains of increasing size; (2) absolute energies of linearly fused benzene chains (acenes); and (3) absolute and interaction energies of water clusters (WCs) of increasing size. In addition, the effect of the basis set size on the DLPNO error is investigated for reaction barriers (RBs) of S_N_2 and Diels–Alder (DA) reactions,^38^ as well as for reaction energies (REs) of DA reactions.^38^

## 2 Computational Details

All of the calculations in this work were carried out with a development version of the ORCA^73−75^ program package based on version 5.0. The canonical and DLPNO-CCSD(T) correlation energies were calculated with the default frozen core settings in ORCA.^76^ CBS extrapolations were performed as described in ref (77) (see the INPUT-CBS sheet of the Supporting Information for the details).

### 2.1 Geometries

The coordinates of linear alkane chains of chemical formula C*_n_*H_2*n*+2_ (the number of C atoms, *n* = 1–20) and linear acene chains of chemical formula C_4*m*+2_H_2*m*+4_ (the number of fused benzene rings, *m* = 1–8) were obtained in this study at the B3LYP-D3(BJ) level^78−82^ within the RIJCOSX approximation^83−86^ and are given in the ALKANE-XYZ and the ACENE-XYZ sheets of the Supporting Information. The def2-TZVP basis and the matching auxiliary basis sets^87^ were used.

**Figure 1:**
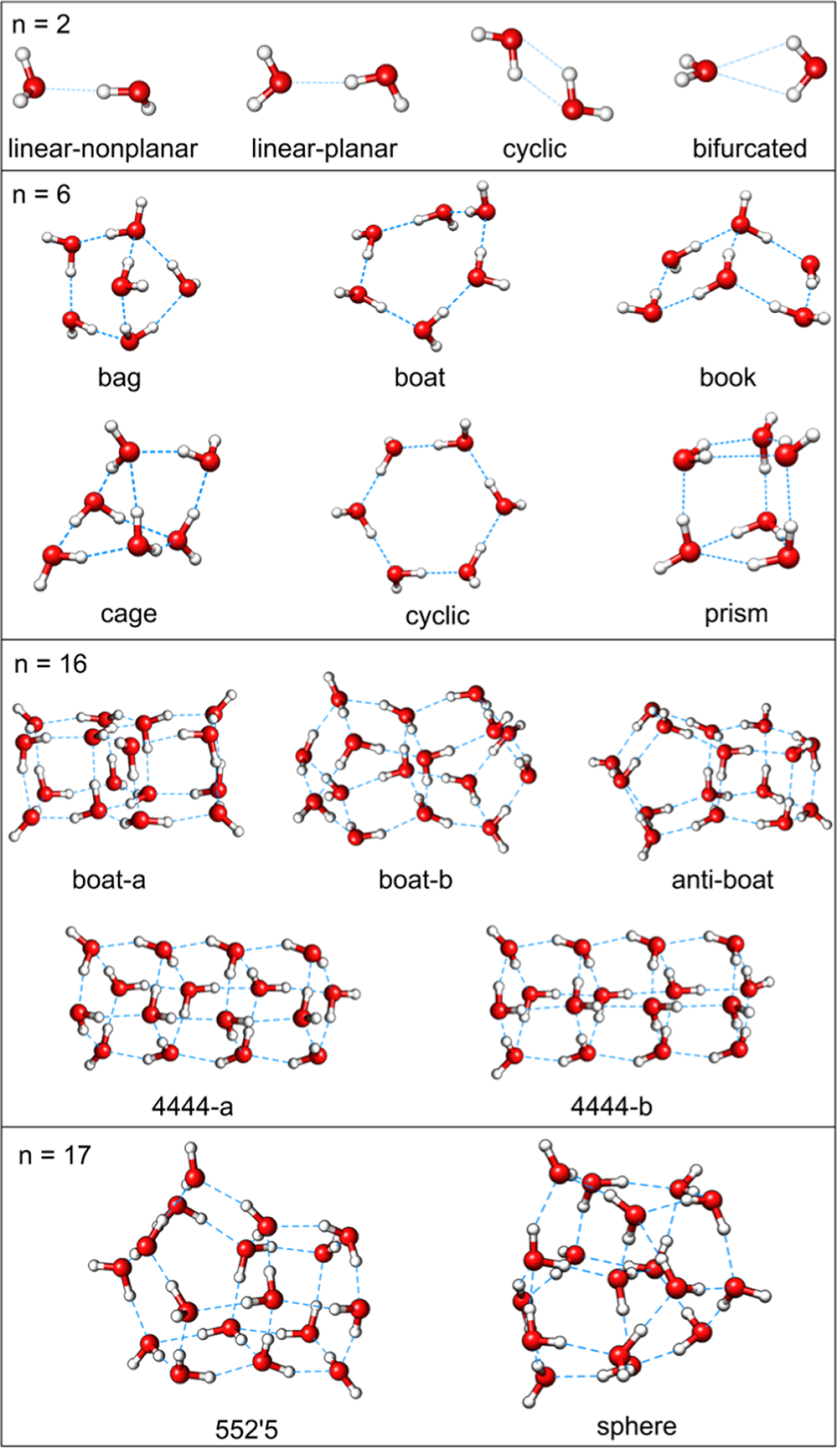
Water clusters (WCs) of chemical formula (H_2_O)*_n_* (*n* = 2, 6, 16, and 17) considered in this work.

The coordinates of WCs (see Figure 1) with chemical formula (H_2_O)*_n_* were taken from ref (88) for *n* = 2 (four conformers), from ref (89) for *n* = 6 (six conformers), and from ref (90) for *n* = 16 (five conformers) and *n* = 17 (two conformers). All coordinates are provided in the WC-XYZ sheet of the Supporting Information.

**Figure 2:**
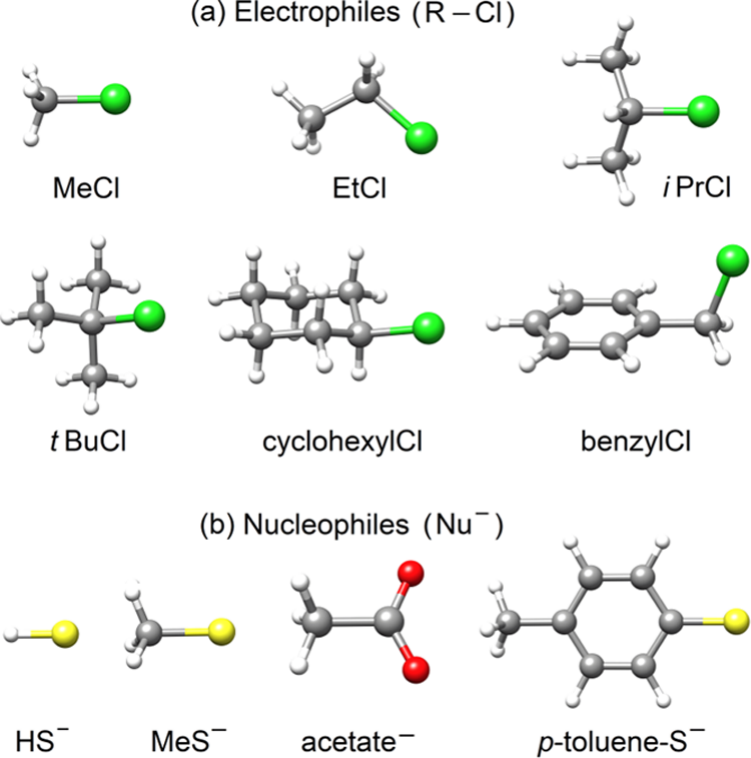
Interacting (a) electrophiles (R–Cl) and (b) nucleophiles (Nu^–^) in the considered S_N_2 reactions (color code: gray, C; white, H; green, Cl; red, O; and yellow, S).

**Figure 3:**
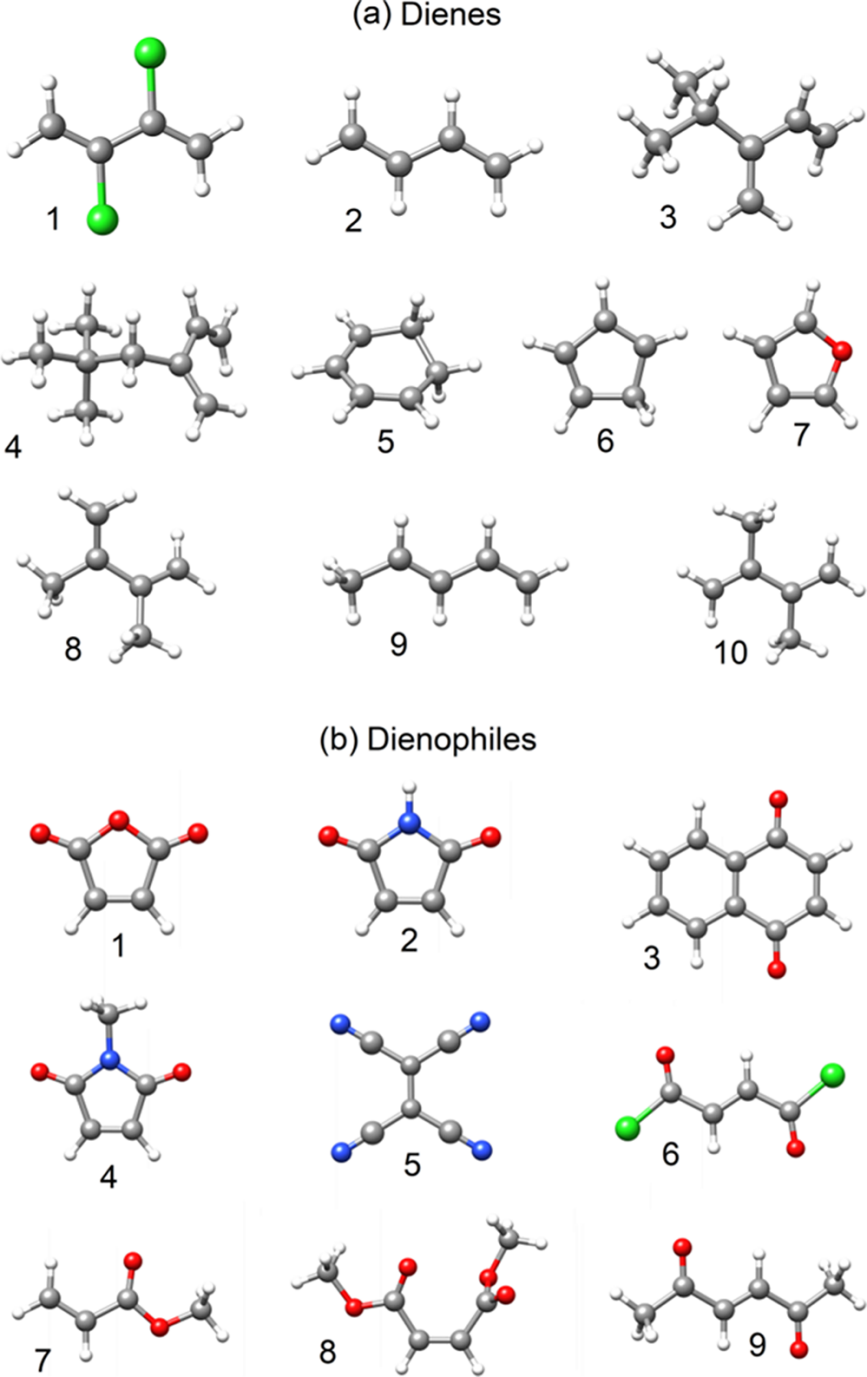
Interacting (a) dienes and (b) dienophiles in the considered Diels–Alder (DA) reactions and their labels (color code: gray, C; white, H; green, Cl; red, O; and blue, N).

The structures for the S_N_2 and DA reaction sets (see Figures 2 and 3) were obtained from ref (38) and are provided in the SN2-XYZ and DA-XYZ sheets of the Supporting Information.

### 2.2 Canonical CCSD(T) References

Canonical CCSD(T) calculations were performed without using the resolution of identity (RI) approximation. For the alkane and acene chains, the cc-pVDZ basis set^91^ was used. For WCs with *n* = 2 and 6, the aug-cc-pVTZ basis set was used.^91^ For WCs with *n* = 16 and 17, canonical CCSD(T)/aug-cc-pVTZ energies were obtained from ref (90). Interaction energies were computed by subtracting from the WC energies the monomer energies at the cluster geometries.

For the S_N_2 RBs and DA RBs and REs, the CCSD(T) results with cc-pVDZ, cc-pVTZ, as well as with CBS(2/3), were taken from ref (38). On a subset of S_N_2 RBs (18 reactions out of 25), we performed the CCSD(T) calculations using the cc-pVQZ basis set and also employed CBS(3/4) extrapolation.

### 2.3 DLPNO-CCSD(T) Calculations

For each molecular set, DLPNO-CCSD(T) calculations were performed with the same basis sets as the parent CCSD(T) calculations described in Section 2.2.

DLPNO calculations exploit the resolution of identity (RI) approximation and hence need an auxiliary “/C” basis set. In this study, unless stated otherwise, the automated auxiliary basis set construction module of ORCA (the so-called “autoaux”) was used to generate conservative /C basis sets with maximum possible angular momentum^92^ (abbreviated as “autoaux-max”, see the input file of these calculations in the INPUT-CBS sheet of the Supporting Information). The effect of the size of the /C basis sets on the RI error was assessed on water dimer, propane, and benzene for the cc-pV*n*Z and aug-cc-pV*n*Z basis sets (*n* = D, T, and Q) and it is discussed in Section 3.1. It was found that, if conservative auxiliary basis sets are used, the RI error is essentially negligible.

The relationship between the basis set superposition error (BSSE) and the DLPNO error was also investigated for interaction energies in WCs. It was found that the DLPNO error is the same for BSSE-corrected and BSSE-uncorrected interaction energies (see the WC sheet of the Supporting Information).

In this manuscript, the (*T*) contribution to the DLPNO-CCSD energetics is obtained using the accurate iterative (*T*_1_) algorithm.^43,93^ The results obtained using the approximate (*T*_0_) correction^15^ are provided for each molecular set in the Supporting Information. Importantly, (*T*_0_) results show a significant error for large systems. This is an inherent error of the (*T*_0_) approximation, which is not related to the truncation of the PNO space and that has already been discussed elsewhere.^43,93^

DLPNO-CCSD(T) calculations were performed with TightPNO settings but using two different *T*_CutPNO_ values, i.e., 10^–7^ (TightPNO default) and 10^–6^. They were then used to obtain CPS(6/7) extrapolated results.

## 3 Results and Discussion

### 3.1 Resolution of Identity Error

Table 1 lists the RI error associated with the DLPNO-CCSD(T)/TightPNO correlation energy of the water dimer (linear-nonplanar conformation, see Figure 1) obtained using different combinations of basis sets and auxiliary /C bases. The error is computed relative to the DLPNO-CCSD(T)/TightPNO results obtained using the same basis set and conservative /C bases (cc-pV6Z/C and aug-cc-pV6Z/C for cc-pV*n*Z and aug-aug-cc-pV*n*Z, respectively).

**Table 1:** RI Error in the DLPNO-CCSD(T)/TightPNO Absolute Correlation Energy (kcal/mol) of the Water Dimer for Different Combinations of Basis Sets and Auxiliary /C Bases

	cc-pV*n*Z			aug-cc-pV*n*Z		
	*n* = D	*n* = T	*n* = Q	*n* = D	*n* = T	*n* = Q
(aug-)cc-pVDZ/C	–0.09			–0.09		
(aug-)cc-pVTZ/C	0.01	–0.12		0.00	–0.11	
(aug-)cc-pVQZ/C	0.00	–0.01	–0.10	0.00	–0.01	–0.10
(aug-)cc-pV5Z/C	0.00	0.00	–0.01	0.00	0.00	–0.01
autoaux^a^	–0.06	–0.06	–0.05	–0.06	–0.07	–0.05
autoaux-max^a^	–0.01	0.00	0.00	0.00	0.00	0.00

a The /C basis sets constructed using the autoaux scheme are abbreviated as (i) autoaux, with matching angular momentum (*n*) of the parent basis and (ii) autoaux-max, with maximum angular momentum.

Correlation energies with matching (aug-)cc-pV*n*Z/C and (aug-)cc-pV*n*Z basis sets, as well as with matching autoaux, show relatively large RI errors. In contrast, the RI error obtained with larger /C basis sets, including autoaux-max, is essentially zero. Similar results were found for propane and benzene, as detailed in the RI-ERROR sheet of the Supporting Information. In the following, all DLPNO-CCSD(T) calculations were performed with the largest autoaux-max auxiliary basis set. Hence, the RI error is expected to be essentially negligible compared to the error associated with the truncation of the PNO space.

### 3.2 DLPNO Error for Linear Alkane and Acene Chains

**Figure 4:**
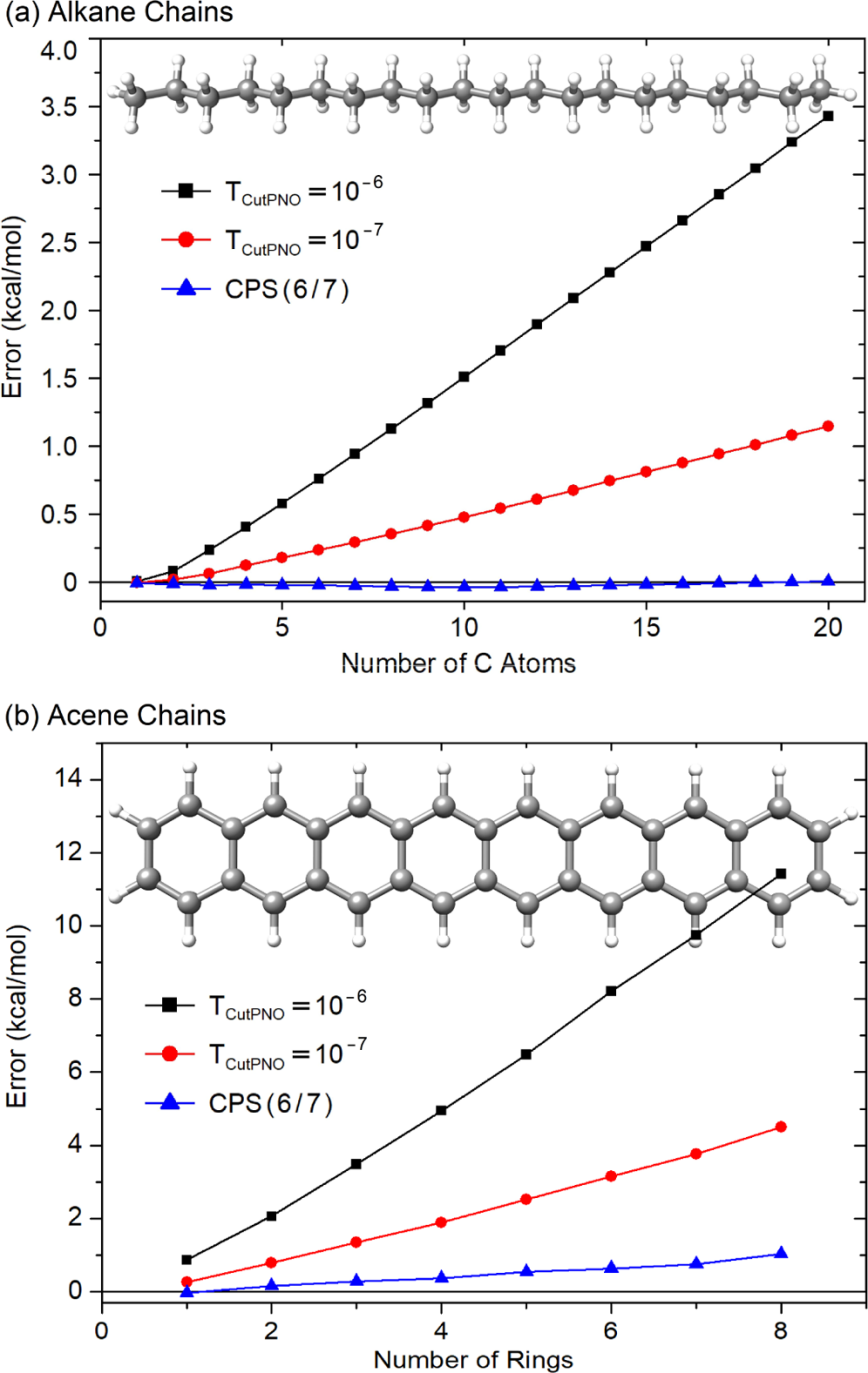
Error in the absolute DLPNO-CCSD(T)/TightPNO/cc-pVDZ energies (kcal/mol) with different *T*_CutPNO_ settings relative to the corresponding canonical CCSD(T)/cc-pVDZ absolute energies for linear (a) alkane chains of chemical formula C*_n_*H_2*n*+2_ (number of carbon atoms, *n* = 1–20) and (b) acene chains of chemical formula C_4*m*+2_H_2*m*+4_ (number of fused benzene rings, *m* = 1–8).

For linear alkane chains (C*_n_*H_2*n*+2_, *n* = 1–20), the variation of the DLPNO error in the absolute DLPNO-CCSD(T)/TightPNO/cc-pVDZ energies with the number of carbon atoms (*n*) is shown with *T*_CutPNO_ = 10^–6^ and 10^–7^ as well as with CPS(6/7) in Figure 4a (see the ALKANE sheet of the Supporting Information for details). For linear acene chains (C_4*m*+2_H_2*m*+4_, *m* = 1–8), the DLPNO error obtained using the same thresholds and basis sets is shown as a function of the number of fused benzene rings (*m*) in Figure 4b (see the ACENE sheet of the Supporting Information for details).

The DLPNO error shows the expected linear increase with increasing number of carbon atoms of alkane chains and benzene rings of acene chains when truncated PNO spaces (*T*_CutPNO_ = 10^–6^ and 10^–7^) are used. The error is effectively reduced upon going from *T*_CutPNO_ = 10^–6^ to *T*_CutPNO_ = 10^–7^ but still increases with the system size noticeably. Remarkably enough, at the estimated CPS(6/7) limit, the error relative to canonical CCSD(T) calculations becomes essentially zero for alkane chains irrespective of the system size. For acene chains, the error associated with the CPS(6/7)-extrapolated results demonstrates an approximatively linear dependence with the system size but with a much smaller slope than *T*_CutPNO_ = 10^–6^ and *T*_CutPNO_ = 10^–7^ results. In fact, CPS(6/7) extrapolation reduces the DLPNO error 4–5 times compared with the default TightPNO settings. Quantitatively speaking, with *T*_CutPNO_ = 10^–6^, *T*_CutPNO_ = 10^–7^, and CPS(6/7), the DLPNO error in the absolute energies is 3.426, 1.145, and 0.004 kcal/mol, respectively, for the largest alkane chain (*n* = 20). It is 11.42, 4.50, and 1.04 kcal/mol, respectively, for the largest acene chain (*m* = 8). This finding demonstrates that the local error in these systems originates to a large extent from the truncation of the PNO space, and hence, it can be diminished using our CPS extrapolation technique. It is also worth mentioning that, by construction, the DLPNO error is positive, meaning that the canonical CCSD(T) limit is approached from below upon tightening the PNO threshold.^6−14^

### 3.3 DLPNO Error for Three-Dimensional Water Clusters

For the set of WCs (Figure 1), the DLPNO error in the DLPNO-CCSD(T)/TightPNO/aug-cc-pVTZ absolute and interaction energies is given in Table 2 with *T*_CutPNO_ = 10^–6^ and 10^–7^ as well as with CPS(6/7). In all cases, the error is obtained positive for each (H_2_O)*_n_* structure (*n* = 2, 6, 16, and 17), and it is given as an average over 4, 6, 5, and 2 conformers, respectively. The energies obtained for the individual conformers are given in the WC sheet of the Supporting Information.

**Table 2:** Mean Error in Absolute Energies (kcal/mol) as well as the Mean Error (kcal/mol) and the Mean Percent Error of Interaction Energies Calculated at the DLPNO-CCSD(T)/aug-cc-pVTZ Level for the (H_2_O)*_n_* Clusters (*n* = 2, 6, 16, and 17) with Different *T*_CutPNO_ Settings Relative to the Corresponding Canonical CCSD(T)/aug-cc-pVTZ Energies

	*n* = 2	*n* = 6	*n* = 16	*n* = 17
	Mean Error for Absolute Energies^a^			
*T*_CutPNO_ = 10^–6^	0.02	0.86	4.05	5.41
*T*_CutPNO_ = 10^–7^	0.06	0.50	2.24	2.66
CPS(6/7)	0.08	0.32	1.33	1.40
	Mean Error for Interaction Energies			
*T*_CutPNO_ = 10^–6^	0.11	1.14	4.80	5.63
*T*_CutPNO_ = 10^–7^	0.05	0.48	2.17	2.44
CPS(6/7)	0.02	0.14	0.86	0.84
	Mean Percent Error for Interaction Energies			
*T*_CutPNO_ = 10^–6^	2.48	2.34	2.71	2.97
*T*_CutPNO_ = 10^–7^	1.15	0.97	1.23	1.29
CPS(6/7)	0.48	0.29	0.49	0.44

a The error for individual water molecules in the clusters is roughly constant and on average equals to −0.047, 0.004, and 0.029 kcal/mol with *T*_CutPNO_ = 10^–6^, *T*_CutPNO_ = 10^–6^, and CPS(6/7), respectively.

As seen in Table 2 and consistent with the results obtained for alkane and acene chains, the error increases significantly with the number of interacting water molecules when truncated PNO spaces are used. However, it reduces by enlarging the PNO space and finally becomes very small for all systems at the estimated CPS(6/7) limit. CPS(6/7) extrapolation reduces the DLPNO error obtained with TightPNO settings by 1.6–1.9 times (for *n* = 6 and larger) for absolute energies and by about 3 times (for all systems) for interaction energies. More specifically, with CPS(6/7) extrapolation, MAEs for interaction energies are 0.02, 0.14, 0.86, and 0.84 kcal/mol with *n* = 2, 6, 16, and 17, respectively. The corresponding mean percent errors are just 0.48, 0.29, 0.49, and 0.44% of the canonical CCSD(T)/aug-cc-pVTZ references. Therefore, the percent errors are small and roughly constant along the entire series. This finding confirms on WCs that the local approximation error can be diminished to a large extent using extrapolation techniques, which leads to accurate energies for large systems.

### 3.4 Basis Set Dependence of the DLPNO Error

As discussed in a previous report,^45^ the DLPNO error for the W4–11 set of atomization energies, the YBDE18 set of ylidic bond dissociation energies, and the S66 set of noncovalent interactions does not increase with the increase of the basis set size (aug-cc-pVnZ, *n* = D, T, and Q). In contrast, for the YBDE18 and S66 sets, the DLPNO error obtained decreases with the basis set size irrespective of the *T*_CutPNO_ threshold used. To further assess the dependence of the DLPNO error on the size of the basis set, we consider here RBs of S_N_2 reactions and RBs and REs of the DA reactions previously used by Sandler et al.^38^ as an illustrative example of the system-size dependence of the local approximation error.

**Figure 5:**
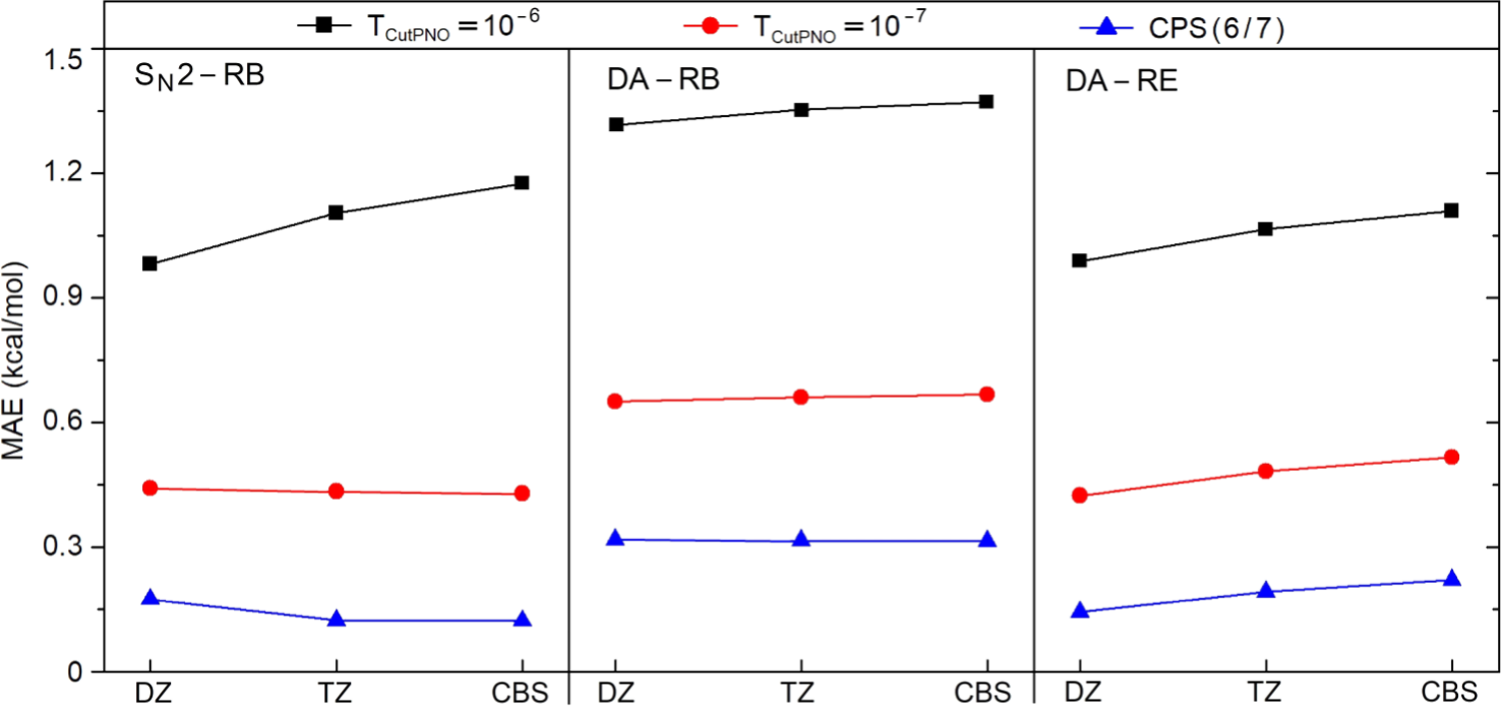
Basis set size dependence (cc-pV*n*Z, *n* = D and T) of the DLPNO-CCSD(T) error (kcal/mol) in reaction barriers (RBs) and reaction energies (REs) of the S_N_2 and Diels–Alder (DA) reactions for the entire sets with different sizes of the PNO space. For each basis set, the error is computed using canonical CCSD(T) energies with the same basis set as a reference.

The MAEs obtained for the entire benchmark sets with cc-pVDZ and cc-pVTZ basis sets, as well as with their CBS(2/3)-extrapolated values, are shown in Figure 5 (see the SN2 and DA sheets of the Supporting Information for the individual energies).

For these reactions, CPS(6/7) extrapolation reduces the MAEs by 2–4 times with respect to TightPNO settings for all of the basis sets tested, consistent with the results mentioned above. With the smallest PNO space considered, i.e., with *T*_CutPNO_ = 10^–6^, the MAE increases very slightly as the basis set size is enlarged. However, with the default TightPNO settings (*T*_CutPNO_ = 10^–7^) as well as at the CPS limit, the MAE variations with the basis set size are negligibly small. Therefore, the error is not much dependent on the basis set size with sufficiently large PNO spaces. Quantitatively speaking, the DLPNO MAEs with CPS(6/7) are

(i) 0.17, 0.12, and 0.12 kcal/mol for DZ, TZ, and CBS(2/3), respectively, for the S_N_2-RB set.
(ii) 0.32, 0.32, and 0.31 kcal/mol for DZ, TZ, and CBS(2/3), respectively, for the DA-RB set.
(iii) 0.14, 0.19, and 0.22 kcal/mol for DZ, TZ, and CBS(2/3), respectively, for the DA-RE set.

**Figure 6:**
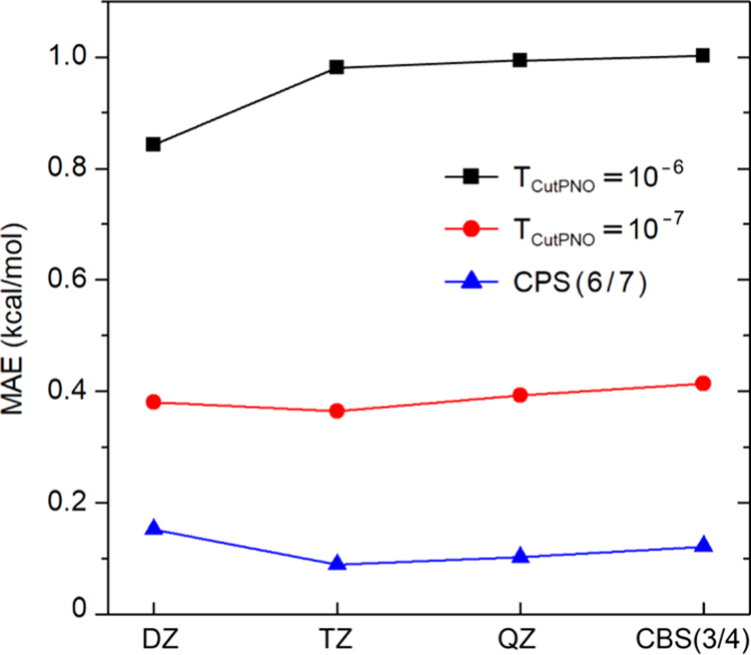
Basis set size dependence (cc-pV*n*Z, *n* = D, T, and Q) of the DLPNO-CCSD(T) error (kcal/mol) in reaction barriers of the subset of S_N_2 reactions with different sizes of the PNO space. For each basis set, the error is computed using canonical CCSD(T) energies with the same basis set as a reference.

For larger basis set sets, i.e., cc-pVQZ and CBS(3/4), canonical CCSD(T) calculations are not feasible for the entire benchmark set. Hence, we defined a subset containing 18 RBs of S_N_2 reactions for which canonical CCSD(T)/cc-pVQZ calculations are still affordable (see the Supporting Information for details). The MAEs obtained for this subset are shown in Figure 6.

For this subset, MAEs of CPS(6/7) with DZ, TZ, QZ, and CBS(3/4) are 0.15, 0.09, 0.10, and 0.12 kcal/mol, respectively. Hence, CPS(6/7) extrapolation clearly provides excellent results with all basis sets.

## 4 Conclusions

CPS(6/7) extrapolation generally reduces the PNO truncation error significantly, which allows us to approach quantitative accuracy in the calculation of relative energies of very large systems. Applications of this approach in host–guest interactions and biological ligand systems are currently being investigated in our laboratory.

## Supporting Information Available

Detailed energetics and error analyses for each benchmark set and Cartesian coordinates of all structures considered (XLSX)
